# Understanding How Mild Traumatic Brain Injury Impacts the Career and Independence of Young Adults

**DOI:** 10.1177/08977151251362109

**Published:** 2025-08-13

**Authors:** Juliet Haarbauer-Krupa, Jason Barber, Nancy Temkin, Lindsay D. Nelson, Tracey Wallace, Nathan Barnett

**Affiliations:** ^1^ Division of Injury Prevention, Centers for Disease Control and Prevention, National Center for Injury Prevention and Control, Atlanta, Georgia, USA.; ^2^ Department of Neurological Surgery, University of Washington, Seattle, Washington, USA.; ^3^ Biostatistics and Rehabilitation Medicine, Department of Neurological Surgery, University of Washington, Seattle, Washington, USA.; ^4^ Departments of Neurosurgery and Neurology, Medical College of Wisconsin, Milwaukee, Wisconsin, USA.; ^5^ Shepherd Center, Complex Concussion Clinic, Atlanta, Georgia, USA.; ^6^ Shepherd Center, Acquired Brain Injury Rehabilitation, Atlanta, Georgia, USA.; ^7^ Department of Rehabilitation Medicine, Emory University, Atlanta, Georgia, USA.

**Keywords:** mild traumatic brain injury, young adults, outcomes

## Abstract

Research on mild traumatic brain injury (mTBI) and its impact on young adults is limited, despite this being an important time in their lives to work toward independence and career development. We analyzed data on 663 persons aged 17–29 years old with mTBI (i.e., TBI with Glasgow Coma Scale scores 13–15) and 170 controls who did not experience an injury from the multicenter, Transforming Research and Clinical Knowledge in TBI study. We assessed participants with mTBI, subdivided into those with computed tomography (CT) evidence of TBI (CT+) and those without (CT−), at 12 months post-injury with measures to examine symptom persistence, work and school status, and functional outcomes. Results indicated differences between mTBI and control participants related to return-to-work, return-to-school, and persistent symptoms. Those in the mTBI group were more likely to experience adverse symptoms and detrimental functional effects compared with controls at 12-months post-injury. However, other factors that may not have been measured could have contributed to these outcomes. Young adults are in a transition period where they are working to achieve independence and to establish careers; however, if they sustain a TBI, they, their families, and their medical providers may not understand how the injury contributes to their outcomes, and they may also have limited experience in seeking resources for care. Outcomes for mTBI could also disrupt their career and life trajectories, making this an important area for further investigation and intervention.

## Introduction

Young adults, especially those under 25, are one of the groups most frequently affected by traumatic brain injury (TBI).^1^ Although experiencing a TBI at any age can have significant consequences, sustaining this injury during young adulthood can significantly impact the trajectory of academic, career, and social development^2^ as well as participation in the community.^3^ Even symptoms from a mild TBI (mTBI; i.e., TBI with Glasgow Coma Scale [GCS] score 13–15) can persist at 12 months post-injury.^4–6^ Common symptoms can include headaches, nausea, vision changes, poor activity tolerance, psychiatric symptoms, and impaired balance.^7^ In addition, research on mTBI suggests those with abnormal imaging findings correlate to more significant injury to the brain, warranting further medical intervention and potentially increasing the likelihood of more pronounced symptoms compared with cases without such findings.^8–12^ Improved awareness of outcomes and care targeting this age group is needed given the relatively high rate of mTBI in young adults and the potential risk for loss of productive years and impaired ability for independent living.^8^

Studies of adults with TBI have generally examined a wide age range that covers the working adult lifespan through the post-retirement period.^13–15^ Younger adults are in the developmental period between adolescence and full adulthood called emerging adulthood, which is marked by increasing independence in managing life, making decisions, and seeking resources.^15,16^ Persons in this age group may have less experience in accessing support and may also have less developed coping skills, which may make them particularly vulnerable when facing new challenges such as mTBI.^16,17^ However, younger adults have not been examined in detail to understand how an mTBI can impact them when establishing a career and lifestyle.

A few small-scale studies have described challenges faced by younger adults returning to school after an mTBI^18–22^; however, none have examined the prevalence of such challenges, the impact on career, nor their long-term trajectories. Further, while mTBI has been associated with substantial longer-term employment consequences for some persons,^23^ the impact on the young adult population has not been examined. Understanding the effect of mTBI on life outcomes in this age group specifically would be helpful because many young adults are amidst transitions in school, career, and lifestyle.

This study characterizes the 12-month outcomes of young adults (ages 17–29) who were seen in the U.S. level 1 trauma centers with mTBI and compares their outcomes with an uninjured control group. We selected this age range as it is when people are transitioning from adolescence to adult lives. The primary outcomes of interest were postconcussion symptoms, work and school status, physical functioning and mental health, and quality of life at 12 months post-injury. Secondary analyses included follow-up care access for the mTBI group.

## Materials and Methods

### Participants

We analyzed data from the Transforming Research and Clinical Knowledge in Traumatic Brain Injury (TRACK-TBI) study involving a prospective cohort of patients with mTBI presenting to 18 level 1 U.S. trauma centers. The institutional review board at each site approved TRACK-TBI study protocols, which were inclusive of secondary data analysis using TRACK-TBI. Participants with mTBI were enrolled from March 2014 and July 2018 and within 24 h of injury in the hospital and followed for at least 12 months post-injury. To be included in the current article, participants had to be between the ages of 17 and 29, have a GCS score of at least 13 upon admission to the enrolling hospital, and have computed tomography (CT) information recorded in the database.

Uninjured friend and family controls were enrolled from August 2017 to September 2019 and followed at comparable time intervals. Inclusion and exclusion criteria for the TRACK-TBI study have been previously reported.^24^ We compared participants with positive intracranial imaging findings (CT+) and those without imaging findings (CT−) to controls. The control group consisted of persons who had not had a TBI in the past year, were recommended by other TRACK-TBI participants, and were similar in age and sex to the TBI participants.

### Outcomes

Measures that contributed to understanding returning to work and school, symptoms, and functional outcomes were selected. The Glasgow Outcome Scale-Extended (GOSE) score is a measure of the association between traumatic injuries and diverse aspects of daily functioning^7,25^ that includes return-to-work and is commonly used as the primary outcome measure in TBI studies. The possible range of scores is from 1 (dead) to 8 (upper good recovery), with less than 8 reflecting some degree of functional limitation after injury. The scale is completed via an interview with patients or proxies.

Respondents were asked to report new injury-related dependence or difficulties (including worsening of any pre-existing problems) in major domains of life function: independence within (i.e., activities of daily living) and outside (e.g., shopping, travel) the home, work, social/leisure activities, family or friend relationships, and other injury-related symptoms or problems affecting daily life.

The work section classifies participants as having no deficit, reduced work capacity, or limited/unable (only able to work in a sheltered workshop or noncompetitive job or currently unable to work). Return-to-school was not directly collected in TRACK, so for this analysis, it was inferred based on all responses and comments regarding student status from the baseline and follow-up interviews. All such decisions were made by one person on a case-by-case basis without knowledge of each subject’s injury group (CT+, CT−, Friend Controls). When the subjects had graduated since the injury or were on summer break at the time of the interview, they were counted as having returned to school. If a subject mentioned “TBI,” “injury,” or “hospital” when describing their circumstances for not being in school, they were counted as not having returned due to the TBI. This outcome is presented descriptively using four categories and analyzed statistically as a binary outcome. Another measure, the Short Form 12-Item Health Survey Version 2 (SF-12V2),^26,27^ assesses life quality from the patient’s perspective and has both a physical and mental health component with higher scores indicating better health. The Rivermead Post Concussion Symptoms Questionnaire was used to record self-reported TBI-related symptoms and has a possible range of 0–64 with higher scores indicating more severe symptoms.^28,29^

### Statistical analysis

This analysis consisted of participants aged 17–29 years presenting with mTBI (GCS 13–15), separately comparing those with positive CT finding and those with negative CT findings to an uninjured control group of similar ages. We quantified differences in baseline characteristics using standardized mean differences (Table 1). We assessed differences in outcomes (return to both work and school) at 12 months for statistical significance using regression modelling. Inverse probability weighting was used to minimize the impact of covariate imbalance, with any remaining imbalance to be addressed using direct covariate adjustment. Rank regression was used for modelling return-to-work as an ordinal variable and Rivermead as a continuous variable. Log-binomial regression was used to model return-to-school as a binary variable, and linear regression for the SF-12 mental and physical components (Table 2). We evaluated differences in individual Rivermead symptoms using nonparametric rank regression and reporting standardized effect sizes. We also ran a regression analysis on the raw Rivermead scores as a sensitivity analysis to understand the magnitude of these effects sizes on the original Rivermead scale (Table 3). Differences in follow-up care through 12-months post-injury were assessed using Mann-Whitney tests for ordinal responses and Fisher’s exact tests for dichotomous responses (Table 4). All outcomes were analyzed using a complete-case approach. A two-sided threshold of *p* < 0.05 defined statistical significance, and primary results were interpreted in the context of multiple comparisons using a 5% false-discovery rate per Benjamini-Hochberg.^26,30^

**Table 1.** Demographic and Clinical Characteristics, 2014–2018

	Total (*N* = 787)	Mild, CT− (*N* = 605)	Mild, CT+ (*N* = 158)	Controls| (*N* = 124)	Mild CT− vs. controls		Mild CT+ vs. controls	
					SMD^*unwt*^	SMD^*wt*^	SMD^*unwt*^	SMD^*wt*^
Age, mean (SD)	23.9 (3.4)	23.8 (3.4)	23.8 (3.4)	24.4 (3.7)	−0.18	0.00	−0.15	−0.07
Female	263 (33%)	189 (37%)	35 (22%)	39 (31%)	0.12	−0.02	−0.21	−0.08
Race								
White	576 (74%)	359 (72%)	124 (79%)	93 (76%)	−0.09	−0.06	0.09	0.05
Black	136 (17%)	103 (21%)	19 (12%)	14 (11%)	0.23	0.04	0.02	−0.02
Asian	32 (4%)	21 (4%)	5 (3%)	6 (5%)	−0.03	0.03	−0.10	−0.04
Other	35 (4%)	16 (3%)	9 (6%)	10 (8%)	−0.28	−0.01	−0.10	−0.02
Hispanic	217 (28%)	134 (27%)	48 (31%)	35 (28%)	−0.04	−0.01	0.05	−0.02
Education years, mean (SD)	13.2 (2.2)	12.9 (2.1)	13.3 (2.1)	13.9 (2.1)	−0.47	−0.09	−0.32	−0.11
Cause of injury								
Motor vehicle collision		238 (47%)	33 (21%)					
Motorcycle collision		43 (9%)	17 (11%)					
Cyclist/pedestrian		76 (15%)	25 (16%)					
Fall		60 (12%)	35 (22%)					
Assault		25 (5%)	21 (13%)					
Other/unknown		63 (12%)	27 (17%)					
Living situation								
Independent living	482 (64%)	298 (62%)	93 (62%)	91 (74%)	−0.29	−0.13	−0.30	−0.11
Living with parent/guardian	264 (35%)	178 (37%)	54 (36%)	32 (26%)	0.20	0.07	0.18	0.04
Other	5 (1%)	2 (0%)	3 (2%)	0 (0%)	0.06	0.06	0.14	0.14
Primary activity								
Work	553 (74%)	354 (74%)	110 (74%)	89 (72%)	−0.04	−0.04	−0.05	−0.03
School	115 (15%)	65 (14%)	24 (16%)	26 (21%)	−0.24	−0.07	−0.16	−0.08
Other	82 (11%)	59 (12%)	15 (10%)	8 (7%)	0.16	0.05	0.10	0.08
Insurance								
Private	432 (58%)	269 (56%)	87 (58%)	76 (63%)	−0.16	0.00	−0.13	−0.04
Medicaid	106 (14%)	76 (16%)	14 (9%)	16 (13%)	0.06	0.07	−0.14	−0.04
Self-pay	188 (25%)	119 (25%)	43 (29%)	26 (21%)	0.06	−0.01	0.14	0.04
Other	24 (3%)	16 (3%)	5 (3%)	3 (2%)	0.04	−0.01	0.04	0.05
ED arrival GCS, mean (SD)		14.8 (0.5)	14.6 (0.7)					
LOC yes/suspected^a^		432 (89%)	129 (87%)					
PTA yes/suspected^a^		373 (82%)	133 (90%)					
Highest level of care								
ED only		176 (35%)	11 (7%)					
Hospital admit		258 (51%)	54 (34%)					
ICU admit		71 (14%)	93 (59%)					
History of TBI^a^								
None	583 (80%)	364 (78%)	123 (84%)	96 (83%)	0.13	0.17	−0.02	0.02
ED only	109 (15%)	72 (15%)	20 (14%)	17 (15%)	−0.02	−0.11	0.03	−0.03
Hospital admit	39 (5%)	32 (7%)	4 (3%)	3 (3%)	−0.20	−0.13	−0.01	0.01
History of neurological problems	98 (12%)	64 (13%)	10 (6%)	24 (19%)	−0.19	0.04	−0.40	−0.13

^a^ Variable values are missing on more than 5% of the relevant subjects.

CT, computed tomography scan of the head; ED, emergency department; GCS, Glasgow Coma Scale; ICU, intensive care unit; LOC, loss of consciousness; PTA, post-traumatic amnesia; SD, standard deviation; SMD, standardized mean difference; UNWT, unweighted; WT, inverse probability weighted for group allocation; TBI, traumatic brain injury.

**Table 2.** Association Between Injury Group and Clinical Outcomes at 12-Months Post-Injury^a^

	Mild, CT− (*N* = 505)	Mild, CT+ (*N* = 158)	Controls (*N* = 124)	Mild CT− vs. controls			Mild CT+ vs. controls		
				Effect size^*b*^ (95% CI)	*p*	*p* ^*MC*^	Effect size^*b*^ (95% CI)	*p*	*p* ^*MC*^
GOSE work (pre-injury workers only)	*N* = 354	*N* = 110	*N* = 89						
No deficit	198 (88%)	67 (97%)	74 (99%)	—	0.003	0.006	—	0.376	0.627
Reduced capacity	22 (10%)	2 (3%)	1 (1%)						
Limited/unable	6 (3%)	0 (0%)	0 (0%)						
Return-to-school (pre-injury students only)	*N* = 65	*N* = 24	*N* = 26						
Still a student (or on break/graduated)	31 (72%)	17 (89%)	16 (84%)	RR = 0.95 (0.69, 1.31)	0.752	0.884	RR = 1.10 (0.85, 1.41)	0.485	0.693
Not a student (due to TBI)	3 (7%)	1 (5%)	0 (0%)	[ref]			[ref]		
Not a student (not due to TBI)	1 (2%)	1 (5%)	3 (16%)						
Not a student (cause unspecified)	8 (19%)	0 (0%)	0 (0%)						
SF-12 V2 Physical Component Score									
Mean (SD)	50.3 (8.7)	52.1 (7.8)	54.8 (5.9)	*B* = −4.6	<0.001	<0.001	*B* = −2.7	0.003	0.006
SF-12 V2 Mental Component Score									
Mean (SD)	49.3 (10.8)	50.1 (10.1)	49.5 (8.5)	*B* = 0.3	0.796	0.884	*B* = 0.1	0.958	0.958
Rivermead Total Score									
Mean (SD)	10.5 (13.6)	9.3 (12.1)	3.5 (6.3)	—	<0.001	<0.001	—	<0.001	<0.001

^a^ Cases with missing outcomes for the mild CT−/mild CT+/control groups were as follows: GOSE work 128/41/14; return-to-school 22/5/7; SF-12 scores 184/53/23; Rivermead 183/52/23.

^b^ Analyses are inverse-probability-weighted for group allocation; statistical significance by rank regression (GOSE work/Rivermead), log-binomial regression (return-to-school), and linear regression (SF-12) with multiple-comparison adjustment by Benjamini-Hochberg (*m* = 10).

CI, confidence interval; CT, computed tomography scan of the head; MC, adjusted for multiple comparisons; GOSE, Glasgow Outcome Scale-Extended; RR, risk ratio; SD, standard deviation; SF-12 V2, 12-Item Short Form Health Survey (version 2); TBI, traumatic brain injury.

**Table 3.** Mean Rivermead Symptom Scores at 12-Months Post-Injury

	Mild, CT− (*N* = 505)	Mild, CT+ (*N* = 158)	Controls (*N* = 124)	Mild CT− vs. controls		Mild CT+ vs. controls	
				β (95% CI)	*p*	β (95% CI)	*p*
Headaches	0.84	0.70	0.31	0.45 (0.22, 0.67)	<0.001	0.45 (0.18, 0.72)	0.001
Dizziness	0.53	0.37	0.18	0.32 (0.08, 0.55)	0.010	0.21 (−0.09, 0.52)	0.168
Nausea	0.30	0.17	0.11	0.17 (−0.05, 0.39)	0.130	0.01 (−0.20, 0.23)	0.918
Noise sensitivity	0.55	0.54	0.10	0.47 (0.25, 0.70)	<0.001	0.54 (0.28, 0.80)	<0.001
Sleep disturbance	0.94	0.76	0.42	0.41 (0.18, 0.64)	<0.001	0.30 (0.03, 0.58)	0.030
Fatigue	0.84	0.70	0.40	0.38 (0.12, 0.64)	0.004	0.29 (0.02, 0.56)	0.035
Irritable	0.82	0.84	0.19	0.49 (0.25, 0.73)	<0.001	0.58 (0.31, 0.85)	<0.001
Depressed	0.57	0.58	0.38	0.21 (−0.06, 0.48)	0.120	0.21 (−0.06, 0.48)	0.131
Frustrated	0.79	0.82	0.39	0.38 (0.12, 0.64)	0.004	0.36 (0.09, 0.63)	0.010
Forgetful	1.08	0.96	0.17	0.71 (0.49, 0.94)	<0.001	0.71 (0.48, 0.93)	<0.001
Poor concentration	0.81	0.84	0.30	0.47 (0.23, 0.71)	<0.001	0.47 (0.23, 0.71)	<0.001
Longer to think	0.91	0.85	0.25	0.60 (0.36, 0.84)	<0.001	0.58 (0.32, 0.84)	<0.001
Blurred vision	0.35	0.26	0.15	0.27 (0.02, 0.52)	0.038	0.18 (−0.10, 0.47)	0.206
Light sensitivity	0.44	0.36	0.10	0.36 (0.15, 0.58)	0.001	0.33 (0.08, 0.58)	0.010
Double vision	0.14	0.07	0.02	0.17 (−0.08, 0.42)	0.141	0.13 (−0.15, 0.42)	0.363
Restless	0.55	0.46	0.09	0.45 (0.23, 0.68)	<0.001	0.45 (0.21, 0.68)	<0.001

Statistical significance by rank regression using inverse-probability-weighted for group allocation. While the standardized effect sizes from the nonparametric modelling are reported in the table, nonstandardized effects sizes from the corresponding parametric models suggest that one standard deviation from the rank-based analysis is comparable with one point on the Rivermead scale. Reported *p* values are unadjusted for multiple comparisons, and all *p* values below 0.036 would remain statistically significant after applying a 5% false-discovery rate per Benjamini-Hochberg (*m* = 32).

CI, confidence interval; CT, computed tomography scan of the head.

**Table 4.** Twelve-Month TBI Outcomes

	Total (*N* = 663)	Mild CT− (*N* = 505)	Mild CT+ (*N* = 158)	*p*
Follow-up care for TBI since injury				
No follow-up care	254 (43%)	220 (49%)	34 (24%)	<0.001
TBI follow-up by physician but no rehab	276 (47%)	186 (42%)	90 (63%)	
TBI follow-up with rehab	62 (10%)	42 (9%)	20 (14%)	
Health care provider for TBI seen since injury	332 (56%)	224 (50%)	108 (75%)	<0.001
Health care provider for other injuries seen since injury	296 (51%)	233 (54%)	63 (45%)	0.081
Any rehab for TBI since injury	62 (10%)	42 (9%)	20 (14%)	0.158
Any INPATIENT rehab for TBI since injury	23 (4%)	11 (2%)	12 (8%)	0.004
Any OUTPATIENT rehab for TBI since injury	46 (8%)	33 (7%)	13 (9%)	0.591
Any rehab for other injuries since injury	124 (21%)	96 (22%)	28 (20%)	0.638
Unknown	{71–88}	{57–70}	{14–18}	

*p* is statistical significance by Mann-Whitney or Fisher’s exact tests (unweighted).

CT, computed tomography scan of the head; TBI, traumatic brain injury.

## Results

### Participant characteristics

The 787 young adults were mostly male (67%) with a mean age (SD) of 23.9 (3.4) years (Table 1). Prior to their injury, 553/750 (74%) were working, 115/750 (15%) were students, and 82/750 (11%) were unemployed, disabled, or homemakers. Persons in all groups seen were primarily White (74%), with 17% Black, 4% Asian, and 4% other race/ethnicities. After applying propensity weighting, the TBI groups were considered reasonably comparable with the friend controls among all relevant baseline characteristics, with all standardized mean differences below 0.20 and most below 0.10.

We looked at injury characteristics in the mTBI cases both with and without intracranial CT abnormalities and found that the CT− group was more likely to be injured as an occupant in a motor vehicle crash ([MVC]; 47% vs. 21%) and to be discharged home from the emergency department (ED) (35% vs. 7%), and was less likely to be admitted to the intensive care unit (14% vs. 59%).

### Outcomes: 12 months follow-up

At 12 months after injury (Table 2), more people in the mild CT− group whose primary activity was work pre-injury reported having a reduced work capacity or being unable to return to work (13% vs. 1% of controls, *p* = 0.006). The mild CT+ TBI group had similar outcomes to controls (3% reduced work capacity). Differences in school outcomes were not statistically significant between groups. Descriptively, 5% and 7% of mTBI participants (CT+ and CT−, respectively) who were students pre-injury reported no longer being in school at 12 months due to the injury. Another 19% of mild CT− mTBI participants were no longer in school for unknown reasons.

Both subgroups of mTBI reported more problems with their physical health than controls (SF-12 physical: 50.3 mild CT−, 52.1 mild CT+, 54.8 controls, *p* < 0.001 for mild CT− vs. controls, *p* = 0.006 for mild CT+ vs. controls), but no differences were seen regarding problems with mental health (SF-12 mental health: 50.1 mild CT−, 49.3 mild CT+, 49.5 controls).

Both mTBI subgroups reported worse problems with related symptoms 12 months after injury compared with uninjured controls (Rivermead Total Score 10.5 mild CT−, 9.3 mild CT+, 3.5 controls, each *p* < 0.001 vs. controls). Symptoms were wide-ranging (Table 3), with the mild CT− group reporting significantly more problems with 13 of the 16 symptoms and mild CT+ with 11 of the symptoms. The largest estimated effect size was for forgetfulness 0.71 standard deviations.

During the 12 months since their injury, participants with mild CT+ TBI were more likely to have received follow-up care (77% mild CT+, 51% mild CT−, *p* < 0.001) and more likely to have had inpatient rehab (8% mild CT+, 2% mild CT−, *p* = 0.004) (Table 4). Differences in outpatient rehab for TBI, any rehab for TBI, seeing a health care provider for other injuries or receiving any rehab for other injuries did not differ significantly between the two mTBI groups.

## Discussion

Research on young adults who experience mTBI during their education, career, and independence transition is limited. To our knowledge, this is the first study to examine this young adult population, particularly the 17–29 age group, who visited the ED for an mTBI and follow-up 12 months later. Having reduced work capacity or being unable to work since study enrollment was more common among participants with CT− mTBI (13%) than uninjured friend controls (1%). This rate was 3% among participants with CT+ mTBI, which was not statistically significant compared with controls. In this examination comparing those with and without imaging findings, we do not have a clear explanation about this difference, in part because we were not able to examine all environmental variables. Participants with CT− mTBI reported less follow-up care after mTBI, which may partially explain this difference.

Differences in school outcomes were not statistically significant between injury and control groups, and even though the sample sizes are too small to make useful inferences, it is noteworthy that three of the four mild CT− students who reported on their perception of why they were not in school attributed it to their TBI. Additionally, participants with mTBI reported poorer physical function and higher levels of neurobehavioral symptoms as indicated on the Rivermead compared with controls. In contrast, emotional functioning was not different between groups. This is particularly striking because prior work has shown that persistent symptoms following mTBI are often related to psychological factors.^25,31,32^

This study may underestimate the impact of mTBI on work and school function, as the outcomes collected are not sensitive to subtle difficulties. For example, persons with work hours that are reduced (vs. pre-injury) by less than 10% or who can do their former work duties but find it more difficult would be marked as “no deficit” on the GOSE work item. Similarly, while we know the percentage of participants who could not remain in school, we do not know the number who remained but experienced significant difficulties and perhaps adjusted their career trajectory/school focus to accommodate their symptoms. In addition to the increased statistical power for the work versus school outcome, we may also see group differences in work but not school because the latter may provide greater support for those who experience longer, persisting symptoms.

High school students are covered by the Individuals with Disability Act (IDEA), which requires schools to address individual needs if a TBI is reported.^33^ Those enrolled in colleges have dedicated Disability Resource Centers (DRCs). However, policies for college students do not include school requirements related to IDEA. In college, students who need accommodations can visit the DRC and approach each professor with what they need to learn, although professors are not required to address accommodations. Nevertheless, college students likely have greater control over their school schedule (e.g., can take a lighter class load, schedule classes at times of day when symptoms are less severe, etc.), which may help them return to school. In contrast, work accommodations may be limited by factors such as financial pressure to maintain the same hours and an inability to control schedule/take breaks. Findings from this study show that mTBI could disproportionately disrupt their career and life trajectories, making this an important area for further investigation and intervention. Continuing to examine these details could yield more insight and may show the need to educate employers or to provide young adults with information to appropriately advocate for their requirements at work.

Additional factors related to a young adult lifestyle have also been associated with mTBI. Young adults are more likely to have consumed alcohol prior to experiencing an mTBI.^34^ Interpersonal violence is more common in this age group and occurs more often among patients who have consumed alcohol.^34^ Screening positive for post-traumatic stress disorder (PTSD) at 6 months after mTBI has been associated with concurrent functional disability, postconcussion, psychiatric symptomatology, decreased satisfaction with life, and with reduced visual processing and mental flexibility.^35^ Patients experiencing PTSD reported fewer years of education and had a higher incidence of self-reported pre-injury psychiatric disturbance.^21^ These confounding variables may disproportionately inflate mTBI symptomology for young adults.

More work is needed to improve methods for identifying those at risk for persistent symptoms and poor work outcomes following mTBI. Efforts should also ensure that first-line providers have knowledge of management options and are referring this population for further care, such as rehabilitation, when appropriate. Noteworthy is that, based on a previous study examining these data, only 10% of the mTBI group received rehabilitation within 12 months post-injury.^24,36^ One could question if a return-to-school and return-to-work is reduced for those who have been described as the chronic mTBI population due to patient’s and provider’s incorrect belief that avoiding activities that exacerbate symptoms is the best treatment.^7,25^

Findings from this study, showing that mTBI may have a long-term impact on employment for some individuals, lend support to current efforts to update the mild/moderate/severe TBI classification system. Researchers and clinicians are working toward generating a system with a greater range of clinical and biological markers beyond just GCS, CT imaging, and duration of loss of consciousness.^25^ Emerging adults are still establishing their independence and developing coping skills so they may have limited experience in seeking resources.

### Limitations

The overall trend of better work- and school-related outcomes for CT+ mTBI compared with CT− mTBI could be related to the higher amount of medical follow-up for TBI in the CT+ population. Another reason for the mild CT− group experience of worse outcomes is their likelihood to be injured in MVCs. MVCs are higher velocity injuries, which could result in greater severity/impact. Previous studies on pediatric and adult populations show worse outcomes and a greater likelihood of referral for specialty care following more severe mechanisms of injury such as MVC.^31–32,37^ These issues further highlight the previous point that current systems of TBI classification, which are based on coarse clinical features and do not adequately consider psychosocial and environmental modifiers, may not account for individual differences in long-term effects of TBI.

In addition, this study collected patient-reported information about effects and outcomes, which may be different than health care records. One-third of participants missed the 12-month outcome assessments, raising questions about representativeness. Finally, this article examines young adults who visited level 1 trauma centers after experiencing a TBI, which may not accurately represent all in this age group including those who do not seek care or visit their primary care physician or a concussion specialty clinic.

The population examined was primarily White, which may reflect current data about hospital visits in minority populations. Racial inequities have not been significantly studied among young adults with mTBI. Racial differences might occur because of varied use of the ED based on insurance status. Racial and ethnic minorities are less likely to have medical insurance,^38^ and several reports have shown differences in health care use, both in-hospital treatment and post-hospital rehabilitation care following a TBI.^39–44^ Future research should focus on follow-up care for all who experience mTBI, including racial inequities, because lingering effects can impact employment, school, and community participation.

## Conclusion

A small but important minority of young adults with mTBI have lasting effects on work and school function 1 year later. Additionally, mTBI in young adults is associated with poorer physical function and greater symptom burden at 12 months post-injury compared with uninjured young adults. Young adults who experience mTBI may have lasting effects that can impact their independence and career trajectory. Further research is needed to better understand this population and to learn more about available resources to support them following the injury.

## Abbreviations Used

CI: Confidence Interval
CT: Computed Tomography of the Head
CT+: Computed Tomography of the Head with imaging findings
CT−: Computed Tomography of the Head without findings
DRCs: Disability Resource Centers
FC: Friend Controls
ED: Emergency Department
GOSE: Glasgow Outcome Scale-Extended
GSC: Glasgow Coma Scale
IDEA: Individuals with Disability Act
MC: Adjusted for Multiple Comparisons
mTBI: Mild TBI
PTSD: Post Traumatic Stress Disorder
RR: Risk Ratio
SF-12V2: Short Form 12 Item Health Survey Version 2
LOC: Loss of Consciousness
MVC: Motor Vehicle Crash
SD: Standard Deviation
SMD: Standardized Mean Differences
TRACK TBI: Transforming Research and Clinical Knowledge in TBI
TBI: Traumatic Brain Injury
UNWT: Unweighted Inverse Probability Weighted for Group Allocation
